# A Roadmap for the Student Pursuing a Career in Pediatric Emergency Medicine

**DOI:** 10.5811/westjem.2019.10.44466

**Published:** 2019-12-09

**Authors:** Aaron N. Leetch, Joshua A. Glasser, Dale P. Woolridge

**Affiliations:** The University of Arizona College of Medicine, Department of Emergency Medicine and Pediatrics, Tucson, Arizona

## Abstract

**Introduction:**

Three pathways are available to students considering a pediatric emergency medicine (PEM) career: pediatric residency followed by PEM fellowship (Peds-PEM); emergency medicine residency followed by PEM fellowship (EM-PEM); and combined EM and pediatrics residency (EM&Peds). Questions regarding differences between the training pathways are common among medical students. We present a comparative analysis of training pathways highlighting major curricular differences to aid in students’ understanding of these training options.

**Methods:**

All currently credentialed training programs for each pathway with curricula published on their websites were included. We analyzed dedicated educational units (EU) core to all three pathways: emergency department (ED), pediatric-only ED, critical care, and research. Minimum requirements for primary residencies were assumed for fellowship trainees.

**Results:**

Of the 75 Peds-PEM, 34 EM-PEM, and 4 EM&Peds programs screened, 85% of Peds-PEM and EM-PEM and all EM&Peds program curricula were available for analysis. Average Peds-PEM EUs were 20.4 EM, 20.1 pediatric-only EM, 5.8 critical care, and 9.0 research. Average EM-PEM EUs were 33.2 EM, 18.3 pediatric-only EM, 6.5 critical care, and 3.3 research. Average EM&Peds EUs were 26.1 EM, 8.0 pediatric-only EM, 10.0 critical care, and 0.3 research.

**Conclusion:**

All three pathways exceed pediatric-focused training required for EM or pediatric residency. Peds-PEM has the most research EUs, EM-PEM the most EM EUs, and EM&Peds the most critical care EUs. All prepare graduates for a pediatric emergency medicine career. Understanding the difference in emphasis between pathways can inform students to select the best pathway for their own careers.

## INTRODUCTION

Many physicians have elected to pursue career pathways focused on the care of children in the emergency setting. Focused training toward such a profession began in a non-accredited format in the early 1980s.^1^ In 1987, the American Board of Emergency Medicine (ABEM) and the American Board of Pediatrics (ABP) published guidelines to combined training in their two specialties. Later, the ABP developed a pediatric emergency medicine (PEM) fellowship-training track with ABEM participation, and the first sub-board certification exam was offered in 1992. As a result, there are three distinct training pathways available to medical students considering a career in pediatric emergency medicine (EM): a three-year pediatric residency followed by a three-year PEM fellowship (Peds-PEM); a three- to four-year EM residency followed by a two-year PEM fellowship (EM-PEM); and a five-year combined EM and pediatrics residency (EM&Peds).

All three pathways provide pediatric emergency care training in excess of what is required by the Accreditation Council for Graduate Medical Education (ACGME) training guidelines for both EM and pediatric residencies.^2^–^4^ Peds-PEM and EM-PEM pathways were established in the 1990s by the ABP in conjunction with ABEM. EM&Peds guidelines were first described in a joint position statement by ABEM and ABP originally published in 1987 and were recently updated in 2016.^5^–^6^ The specific requirements for Peds-PEM, EM-PEM and EM&Peds are detailed in Table 1.

Residency and fellowship programs are given autonomy by the ACGME to design their curricula in accordance with the resources of their institutions and the needs of their residents or fellows within approved training guidelines.^2^–^6^ All pathways impart pediatric emergency care expertise but with differences in core training content that lead to a variation in clinical practice. Given the five-to-six-year training commitment, it is crucial that medical students considering a career in pediatric EM understand the nuances of each pathway prior to the National Resident Matching Program submission deadlines. We present a curriculum analysis that aims to elucidate the different clinical trajectories of each pathway and aid in appropriate selection for the individual student’s career goals.

## METHODS

We obtained a list of currently credentialed Peds-PEM, EM-PEM, and EM&Peds programs from the ACGME website in January 2018. Each program’s curriculum was obtained from its official website. Programs with insufficient curriculum or no curriculum posted on their websites were excluded. We analyzed dedicated educational units (EU) regarding time spent in the emergency department (ED) (adult or not specified between adult & pediatric), pediatric-only ED, critical care (including adult medical, trauma, pediatric, and neonatal), and research. These were chosen as they are the most common for comparison purposes and make up the majority of EUs in each of the three pathways. The ACGME considers 12-month/year and 13-block/year EUs to be equivalent. EUs split between two experiences were assigned 0.5 EU to each area. Results were averaged for each of the three training pathways.

**Educational Research Capsule Summary**

What do we already know about this issue?

*There are three pathways toward a career in pediatric emergency medicine (EM), each with its own strengths and limitations*.

What was the research question?

What are the differences between pediatric EM training pathways that students pursuing this career should understand?

What was the major finding of the study?

*The curriculum of each pathway with their noted strengths can be used to guide students toward their ultimate desired careers*.

How does this improve population health?

*Students can identify their career path early in their training toward future careers focused on the care of children in the emergency setting*.

Peds-PEM and EM-PEM graduates were assumed to have fulfilled the minimum ACGME-required EUs during their primary residency and these were added to each fellowship’s totals prior to averaging. These include three EU pediatric-only ED and four EU critical care required to complete a pediatric residency, and five EU pediatric ED and four EU critical care required to complete an EM residency. Dedicated EUs spent in the ED for primary EM residents were assumed to be 21.8 based on the mean number for three-year EM residencies published in 2015 by Stowell et al.^7^ We used Google Sheets (Google, Mountain View, CA) to tabulate and average the totals.

## RESULTS

We identified a total of 113 active programs from the ACGME website (75 Peds-PEM; 34 EM-PEM; 4 EM&Peds). Of these, 64 Peds-PEM (85%) programs, 29 EM-PEM (85%), and 4 Peds-EM (100%) had published curricula on their official websites and were included. Results are tabulated in Table 2.

The EM-PEM training track demonstrated the most overall dedicated ED EUs (35.4) followed by EM&Peds (26.1) and Peds-PEM (20.4). Peds-PEM graduates have the most dedicated pediatric-only ED EUs (20.1) followed by EM-PEM (18.3) and EM&Peds (8.0). EM&Peds graduates have the most dedicated critical care EUs (10) followed by EM-PEM (6.5) and Peds-PEM (5.8). Peds-PEM graduates have the most dedicated research EUs (9.0) followed by EM-PEM (3.3), and EM&Peds (0.3).

## DISCUSSION

As all three pathways offer pediatric emergency training beyond what the primary residencies of EM or pediatrics offer, it is the route taken that will most affect the ultimate career options. The overlapping strengths of each provide an environment for the graduates of each training pathway to gain sufficient experience in both acute and critical care of the pediatric patient. The strengths and potential limitations of each pathway are highlighted in Table 3.

### Pediatrics-Pediatric Emergency Medicine Pathway

The Peds-PEM pathway offers the most overall training in pediatrics with a foundation of ambulatory and inpatient care in the primary residency followed by specialty training in pediatric emergency care during the fellowship. This requires both a residency and a fellowship match. The Peds-PEM route aims to train pediatricians first and then focus them into pediatric emergency physicians through a large amount of time spent in the pediatric ED with a targeted exposure to adult EM as required by the ACGME. There is a heavy focus on dedicated research time compared to the other pathways. Peds-PEM graduates are eligible for the PEM sub-boards co-sponsored by ABEM and ABP. The clinical scope of Peds-PEM is limited to patients <21 years of age, making children’s hospitals or EDs with a high pediatric volume the ideal career for these graduates. However, these age limits are noted to be arbitrary and 21 years is not a firm limit.^8^ Still, much of adult EM will be outside the scope of training and hospital privileges afforded to Peds-PEM graduates. Peds-PEM duration of training is six years without variation as described by the ACGME program requirements for PEM fellowships.^4^

### Emergency Medicine-Pediatric Emergency Medicine Pathway

The EM-PEM pathway offers the most overall training in EM with a foundation of emergency and critical care in the primary residency followed by specialty training in pediatric emergency care during the fellowship. This requires both a residency and a fellowship match. The EM-PEM route aims to train emergency physicians first and then to focus them into pediatric emergency physicians through dedicated pediatric ED time and some subspecialty pediatrics. EM-PEM graduates are eligible for the PEM sub-boards co-sponsored by the ABP and the ABEM. The clinical scope of EM-PEM is all ages, although they are limited to an ED practice setting without the potential for pediatric ambulatory or inpatient medicine. EM-PEM duration of training is between five and six years. This is variable as students may choose to pursue a three- or four-year EM primary residency prior to their two-year fellowship.

### Emergency Medicine and Pediatrics Pathway

The EM&Peds pathway offers the broadest training of all pathways with complete training in both general pediatrics and emergency medicine. This requires only a residency match. The EM&Peds route aims to simultaneously train emergency physicians and general pediatricians, resulting in pediatric emergency physicians. Alhough this pathway has the least amount of pediatric-only ED EUs, the philosophy is to learn procedural skills and acute care principles through EM residency training and complete care of the pediatric patient through ambulatory and inpatient pediatric rotations. The result is not only an exposure to all aspects of pediatric and EM care but also a heavier focus on critical care compared to the other pathways.

EM&Peds graduates are eligible for dual board certification in both EM and general pediatrics but have not been eligible for the PEM sub-board certification since 1998. Some centers, predominately freestanding children’s hospitals that care only for children, consider PEM sub-board certification a prerequisite which may be a limitation for EM&Peds graduates. However, the versatility of EM&Peds training may be a strength to centers that care for both adults and children. Many EM&Peds graduates work in academic, community, or rural centers.^9^ EM&Peds graduates are trained to care for children in ED, ambulatory and inpatient settings. EM&Peds training duration is five years as set by the joint ABP and ABEM agreement.

### Nuances Between Pathways

The EM&Peds physician and the Peds-PEM physician both share the primary pediatric board, allowing eligibility for additional ABP-sponsored fellowship training or shared time as a clinical pediatrician or pediatric hospitalist in addition to their EM practice. Similarly, the EM&Peds physicians and EM-PEM physician share the primary EM board allowing for EM fellowship training potential. Certainly, all of the pathways in pediatric EM provide a background for such physicians to take positions of advocacy and leadership in clinical and academic settings.^10^

There are notable differences in the number of physicians trained through each pathway. A 2006 pediatric study referencing the Future of Pediatric Education II data revealed that at the time there were approximately 1300 ABP-certified Peds-PEM practitioners compared to only 170 ABEM-certified EM-PEM practitioners, a proportion that has likely continued to shift to less representation by EM-PEM physicians.^11^ In 2007, Murray et al. also showed through a survey of PEM fellowship programs that only 5% of entering fellows had an EM primary board background.^12^ More recently, 2018 ABEM data reveals that in 2017, only 40 ABEM-eligible EM-PEM physicians were enrolled in PEM fellowship programs, suggesting that only ~ 20 EM residency graduates enter PEM fellowships annually.^13^

The reasons for this are not clear, although recently the ABEM EM to PEM taskforce has sought to address this difference. One possibility is that EM graduates do not seek to be further specialized as acute care of children is already within their scope of practice.^2^ Centralization of pediatric emergency care may also lead to fewer opportunities for EM-PEM graduates in community EDs, where PEM fellowship training would not necessarily be more advantageous than general EM training alone.^14^ During our research we noted that there were less EM-based PEM fellowships (29) compared to pediatrics-based PEM fellowships (64). Notably, several pediatrics-based programs that published a Peds-PEM curriculum did not publish an EM-PEM curriculum.

Although possibly due to omission from their websites, PEM fellowship programs are not required to accept both pediatrics and EM candidates. This may indicate fewer available fellowship opportunities for the EM-PEM pathway compared to Peds-PEM or that EM-PEM trainees are required to complete three years at that fellowship instead of two.^15^ Lastly, financial differences may contribute to this issue. PEM-fellowship trained physicians traditionally have a lower salary than general emergency physicians.^16^ However, EM&Peds graduates do report making similar salaries to that of their general EM colleages.^9^ We speculate that EM-PEM graduates likely make similar salaries to EM and EM&Peds graduates given their capacity to care for adults. We also speculate that salary is more likely related to the practice setting than the training itself, although the training does in part help determine the practice setting.

There were 48 postgraduate year 1–5 candidates enrolled in EM&Peds programs in the ABEM dataset, making it the second most common pathway chosen. Still the vast majority of pediatric emergency providers are Peds-PEM, making the EM&Peds pathway less well known by comparison. More research might reveal more subtle differences between the specifics of these training pathways as regards specific procedural experience, patient volume, or other metrics.

### Choosing a Pathway

What may be considered a limitation to one student may be a strength to another. An appropriate starting point may be whether the student wishes to care for adults or only children. Should students not wish to care for adults, a Peds-PEM pathway would be most suitable. If students would like to care for adults, the applicant would be directed toward either EM-PEM or EM&Peds. The difference here is eligibility for the PEM sub-boards and general pediatrics exposure for the EM&Peds graduate. EM-PEM graduates are eligible for sub-board certification, which may increase the likelihood for employment in some children’s hospitals or other centers that require subspecialty certification. EM&Peds graduates are no longer eligible for sub-board certification, which can be a deterrent to certain centers. However, EM&Peds graduates have a much broader scope of practice with the potential for more varied career paths including ambulatory and hospital pediatrics to which EM-PEM graduates do not have access. This may be attractive to centers looking to employ a provider in several clinical areas or departments. Students can certainly blaze their own trail within a given pathway but should be aware and well prepared for the path ahead of them.

## LIMITATIONS

Data collected is limited to only those programs with a published online curriculum. By not polling programs directly, this does give an incomplete picture and may have failed to recognize more recent developments in certain programs. However, the authors felt that this approach was similar to that of a medical student researching future career options and was thus appropriately realistic with a relatively large sample size. Confirmation and clarification from programs would increase the overall accuracy of the available data by ensuring only the most recent/updated curriculum was used, and would add more data points by including programs without a publicly published curriculum.

Longitudinal experience was not accounted for in the dataset as only dedicated EUs were included. Similarly, many training programs also incorporate clinical shifts into elective or research time. However, dedicated EUs are what is mandated by the ACGME as well as by the ABP and ABEM for board certification and thus are a better marker of the overall goals of training programs. The exact number of dedicated EUs that Peds-PEM and EM-PEM residents do during residency was estimated. However, all graduates from accredited pediatrics or EM primary residencies are eligible for the PEM fellowship, thus making the minimum number required a reasonable estimation. To our knowledge, a central resource with this depth of analysis and information was not previously available to medical students considering their career choices.

## CONCLUSION

Three training pathways lead to expertise in pediatric emergency medicine although with different career trajectories. Peds-PEM training is ideal for the student who does not wish to care for adults, although clinical career options may be limited to children’s hospitals or EDs with a high enough pediatric volume to sustain the narrower scope of practice. EM-PEM and EM&Peds pathways are similar, although the lack of sub-board eligibility for EM&Peds may be a limitation for clinical careers in centers that require the sub-board certification. The curriculum of each pathway can be used to guide students toward their ultimate desired career. Understanding the characteristics of current available paths will hopefully set students up for success in future careers focused on the care of children in the emergency setting.

## Figures and Tables

**Table 1:** Accreditation Council for Graduate Medical Education (ACGME) curricular requirements for pediatric emergency medicine pathways.

Pathway	Required EUs per ACGME Guidelines	
EM&Peds	General pediatric requirements: 3 pediatric emergency department and acute illness 1 developmental-behavioral 1 adolescent medicine 1 term newborn 5 inpatient pediatrics 2 ambulatory experiences 2 neonatal intensive care 2 pediatric intensive care 7 pediatric subspecialty	Emergency medicine requirements: 4 critical care 5 pediatric emergency department
Peds-PEM	General pediatrics requirements as above plus: 12 pediatric-only emergency department 4 adult emergency medicine, including 1 adult trauma, 1 emergency medical services, 1 toxicology	
EM-PEM	General emergency medicine requirements as above plus: 12 pediatric-only emergency department 4 pediatric training; including 1 ambulatory pediatrics, 1 care of critically ill neonates, 1 care of critically ill children	

*EU*, educational units defined as 1 month or 1 block in a 13-block/year schedule; *Peds-PEM*, pediatrics-pediatric emergency medicine fellowship; *EM-PEM*, emergency medicine-pediatric emergency medicine; *EM&Peds*, combined emergency medicine and pediatrics residency.

**Table 2:** Results of average educational units (EU) in each pathway according to published curricula.

Pathway	Peds-PEM (n=64)	EM-PEM (n=29)	EM&Peds (n=4)
Total ED	20.4 (17 – 34)	35.4 (32.8 – 40.8)	26.1 (23.5 – 31.5)
Pediatric-only ED	20.1 (16 – 31)	18.3 (14 – 22)	8.0 (6 – 10)
Critical care	5.8 (4 – 8)	6.5 (4 –9)	10.0 (8 – 12)
Research	9.0 (1 – 15)	3.3 (1 – 8)	0.3 (0 – 1)

*Peds-PEM*, pediatrics-pediatric emergency medicine fellowship; *EM-PEM*, emergency medicine-pediatric emergency medicine; *EM&Peds*, combined emergency medicine and pediatrics residency; *ED*, emergency department.

**Table 3:** Strengths and potential limitations of training pathways in pediatric emergency medicine.

Characteristics	Peds-PEM	EM-PEM	EM&Peds
Duration of training	6 years	5–6 years	5 years
Training methodology	General pediatric residency with PEM fellowship	EM residency with PEM fellowship	Simultaneous EM and general pediatric residency
NRMP cycles	2	2	1
Training focus by curriculum	Research pediatric ED	General ED Pediatric ED	General pediatrics General ED Critical care
Primary board eligibility	General pediatrics	Emergency medicine	General pediatrics and emergency medicine
PEM Sub-board eligible?	Yes	Yes	No
Can care for adults?	No	Yes	Yes
Ambulatory or hospitalist potential?	Both	Neither	Both

*EM*, emergency medicine; *PEM*, pediatric emergency medicine; *Peds-PEM*, pediatrics-pediatric emergency medicine fellowship; *EM-PEM*, emergency medicine-pediatric emergency medicine; *EM&Peds*, combined emergency medicine and pediatrics residency; *NRMP*, National Residency Match Program.
